# Smartphone based colorimetric approach for quantitative determination of uric acid using Image J

**DOI:** 10.1038/s41598-023-48962-0

**Published:** 2023-12-11

**Authors:** Samar H. Elagamy, Latifa Adly, Mohamed Ahmed Abdel Hamid

**Affiliations:** 1 Department of Pharmaceutical Analytical Chemistry, Faculty of Pharmacy, Tanta University, Tanta, Egypt; 2 Department of pharmaceutical chemistry, Al Salam university, Tanta, Egypt

**Keywords:** Chemistry, Optics and photonics

## Abstract

Recently, significant attention has been directed towards digital image colorimetry DIC using mobile applications or available software programs, which offer the advantage of analyzing samples without the need for sophisticated instruments. One such image processing program is Image J, widely used for obtaining quantitative information from scientific images. Image J could measure the color intensities by quantifying of the RGB (red–green–blue) gray levels across the images of colored substances. These values are correlated to the color intensities through conversion to CMY (cyan–magenta–yellow) values which are proportional to the color intensities. The objective of this study is to develop an innovative analytical method for the quantitative determination of uric acid using Image J for color quantification. Image J was utilized to analyze images captured by smart phone for successive concentrations of uric acid that were previously treated with phosphotungstate to develop a blue color. The proposed method has been applied for determination of uric acid in real urine using standard addition method and the results were compared with UV/VIS spectrophotometry as a reference method. In this research, we will also assess the effectiveness of quantitative analysis using Image J in comparison to a mobile application, namely RGB Color Detector.

## Introduction

In the last few years, there has been a growing interest in incorporating image processing programs and mobile applications APPS into chemical analysis which permits green, fast and cost-effective analysis^1^. These programs and AAPS have been utilized for colorimetry^2,3^, fluorimetry^4^, chemiluminescence^5,6^ and electrochemical analysis^7^. Extensive research has been conducted on digital image colorimetry (DIC) as a versatile technique for both qualitative and quantitative analysis across various sample types including heavy metals^8^, pesticides^9,10^, antibiotics^11^, environmental samples^12^ and food products^13–15^. DIC is based on image acquisition with smartphone and color quantification using mobile APPS (RGB color detector, color meter, PhotoMetrix) or image processing programs (Adobe Photoshop, Image J, Matlab) under appropriate color system. The color quantification data were utilized to obtain analyte concentration^16^. Smartphones are preferable to digital cameras for image acquisition in DIC due to their portability, simplicity in use, significant improvement of camera functions, and the availability of mobile APPS^17,18^.

Image J is an open-source image processing program designed by the national institutes of health NIH for analysis of scientific images. Image J has been extensively used in different biological studies include quantifying cellular and subcellular components^19^. It has also been successfully applied in analysis of various medical images including dental imaging, tumor differentiation, and brain tissue imaging^20–22^. Image J offers several benefits compared to other image processing software such as the accessibility and ease of use of basic built in functions even without prior experience in image analysis. Furthermore, it has the capability to function on a wide range of computer platforms and possesses the ability to handle the majority of commonly used image formats. Image J functions include noise reduction, background subtraction, smoothing, sharpening, contrast manipulation, detection of regions of interest, and quantifying intensities. Additionally, it facilitates measurements of areas, volumes (stacks), distances, and angles^23–25^.

**Figure 1:**
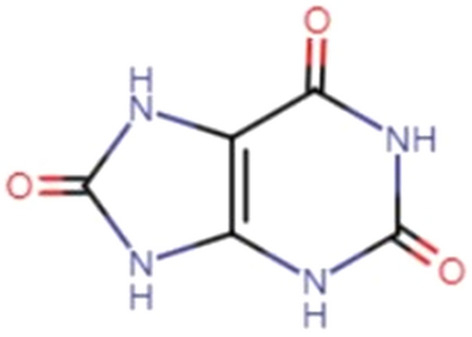
Chemical structure of uric acid.

Uric acid is a product of metabolic breakdown of purine (adenine and guanine) that exist in RNA and DNA Fig. 1. It is a weak acid with a pKa of 5.6 and it is sparingly soluble in water. The concentration of uric acid in serum and urine is an indicator of many renal complications. The normal uric acid concentration in urine is 250–750 mg/day. A high uric acid level (greater than 800 mg/day) can cause uric acid crystallization and deposition in joints, tendons and surrounding tissues and development of other medical conditions such as gout or urinary stones^26,27^. Different chromatographic methods have been developed for determination of uric acid and creatinine in human urine^28–30^. In addition, several colorimetric methods have been recently reported^31–34^. In this study, we have applied DIC using Image J for quantitative determination of uric acid after treatment with phosphotungstate reagent in alkaline medium to develop a blue color. Image J was chosen for color quantitation as it outperforms mobile APPS in its capability to enable precise quantitative analysis, while mobile APPS is primarily suitable for qualitative or semi-quantitative analysis. This work is considered to be the first approach to use Image J as image processing program in DIC and the first investigation of the capabilities of image J in comparison to mobile APPS.

## Materials and methods

### Apparatus

Spectrophotometric measurements have been carried out using a Shimadzu (Japan) UV-1800 PC double beam spectrophotometer with 1 cm Quartz cells. UV probe software was used for spectra displaying and arithmetic calculations.

### Materials

Uric acid, Sodium chloride NaCl, Sodium citrate Na_3_C_6_H_5_O_7_·2H_2_O, Sodium sulfate Na_2_SO_4_, Magnesium sulfate MgSO_4_·7H_2_O, Potassium chloride KCl, Potassium dihydrogen phosphate KH_2_PO_4_ were purchased from Fisher Scientific Company. Phosphotungstate reagent was kindly supplied from biochemistry department, faculty of pharmacy, Tanta university. Protein free urine sample collected over 24 hours was supplied from Elmenshawy hospital after the patient consent.

### Preparation of artificial urine

Artificial urine was prepared by dissolving 1.5 gm Sodium chloride, 0.96 gm Potassium chloride KCl, 1.0982 gm Potassium dihydrogen phosphate, 1.0 gm Sodium citrate, 0.6 gm Sodium sulfate, and 0.1155 Magnesium sulfate in 250 mL distilled water^35^.The pH of the prepared artificial urine was found to be 5.9.

### Determination of uric acid in artificial urine

Uric acid stock solution (30 μg·mL^−1^) was prepared by dissolving 3.0 mg uric acid in diluted artificial urine 10:90 with distilled water DW. Different aliquots (1–5.0 mL) were quantitatively transferred into a series of 10 mL volumetric flask. 3.0 mL of Na_2_CO_3_ aqueous solution (10%) was added to each flask, and they were allowed to stand for 10 minutes. After that, 1.0 mL of follin reagent was added, mixed well using vortex and the volume was completed with DW to give final concentrations of 3.0–15 μg·mL^−1^.

### Determination of uric acid in real urine

10 mL of urine sample was diluted with 90 mL DW and the amount of uric acid in the sample was determined using standard addition method to eliminate the matrix effect through addition of different volumes (0.5, 1.0, 2.0, 3.0 mL) of uric acid standard solution (30 μg·mL^−1^) to 0.5 mL of diluted urine followed by the same procedure for color reaction under Section “Determination of uric acid in artificial urine”. The amount of uric acid in the real urine was calculated from the regression equation and then multiplied by dilution factor of 200:1 to obtain the initial concentration.

### Imaging procedure

**Figure 2:**
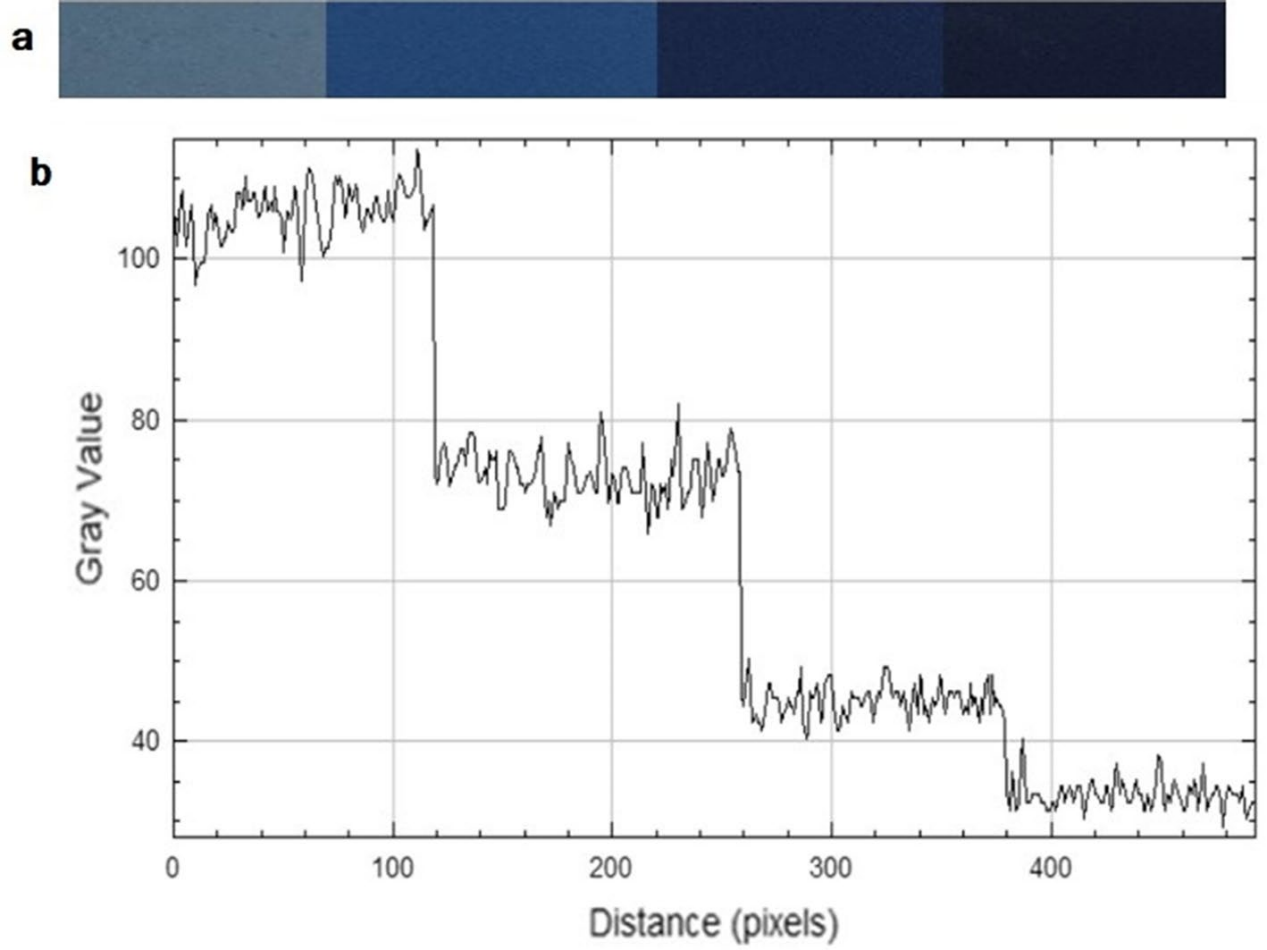
(**a**) The images of successive concentrations of uric acid (**b**)their corresponding gray value plot versus distance.

The serial dilutions of uric acid were placed in glass cuvettes and the images were captured using smart phone (Samsung Galaxy A52; 64 Mpx camera resolution), against white background in an imaging box in order not to be affected by daylight and to improve the signal to noise ratio^2^. The images were cropped to remove blank spaces on all edges and brought together in one image with each segment representing a certain concentration Fig. 2a. The images were saved as TIFF (Tagged Image File Format). The average of the gray values was calculated for each segment.

## Results and discussion

In alkaline medium uric acid reduces phosphotungstate reagent producing tungsten blue color according to the Eq. 1^26^

1 $$ {\text{Uric acid}} + {\text{phosphotungstate}} + {\text{Na}}_{{2}} {\text{CO}}_{{3}} \ldots \ldots \ldots \ldots ....{\text{Allantoin}} + {\text{tungsten blue}} + {\text{CO}}_{{2}} $$

There is a linear relationship between the intensity of blue color and the concentration of uric acid. This was exploited to apply digital image colorimetry DIC using image J for color quantification. The RGB gray values were measured across the images of serial dilution of uric acid using image J and displayed as a plot of gray values versus the pixel positions. The RGB gray value is a numerical representation of the intensity of gray or color in a specific pixel, ranging from 0 (black) to 255 (white). This explains why in Fig. 2b, as depicted, the gray value increases with the reduction in color intensity. To establish a correlation between the RGB gray values and color intensity, they were transformed into complementary CMY (cyan–magenta–yellow) values using the formula CMY = 255 − RGB^36^.

### Optimization of experimental condition

All variables impacting the color reaction between uric acid and phosphotungstate were meticulously optimized to achieve an intense blue color and to maximize the absorbance value, consequently enhancing the method's sensitivity. The quantity of phosphotungstate reagent was subjected to examination, revealing that an optimal volume of 1.0 mL yielded the best results (see Fig. S1). Similarly, the influence of sodium carbonate amount was investigated, and the peak absorbance was attained with 3.0 mL of sodium carbonate (see Fig. S2). Furthermore, an optimum vortex time following the addition of the reagent was found to be 10 min (see Fig. S3).Table 1 summarizes all the factors affecting the color intensity and their optimal values.

**Table 1:** Factors affecting the color intensity.

Factors	Studied range	Effect	Optimum conditions
Volume of phosphotungstate reagent	0.2–2.0 mL	Increasing the volume results in increasing the color intensity followed by subsequent decrease	1.0 mL
Volume of sodium carbonate	1.0–5.0 mL	Increasing the volume results in increasing the color intensity then it remain unchanged	3.0 mL
Vortex time	3–15 min	Increasing the time results in increasing the color intensity then it remains constant	10 min

### Comparison with a mobile application

**Figure 3:**
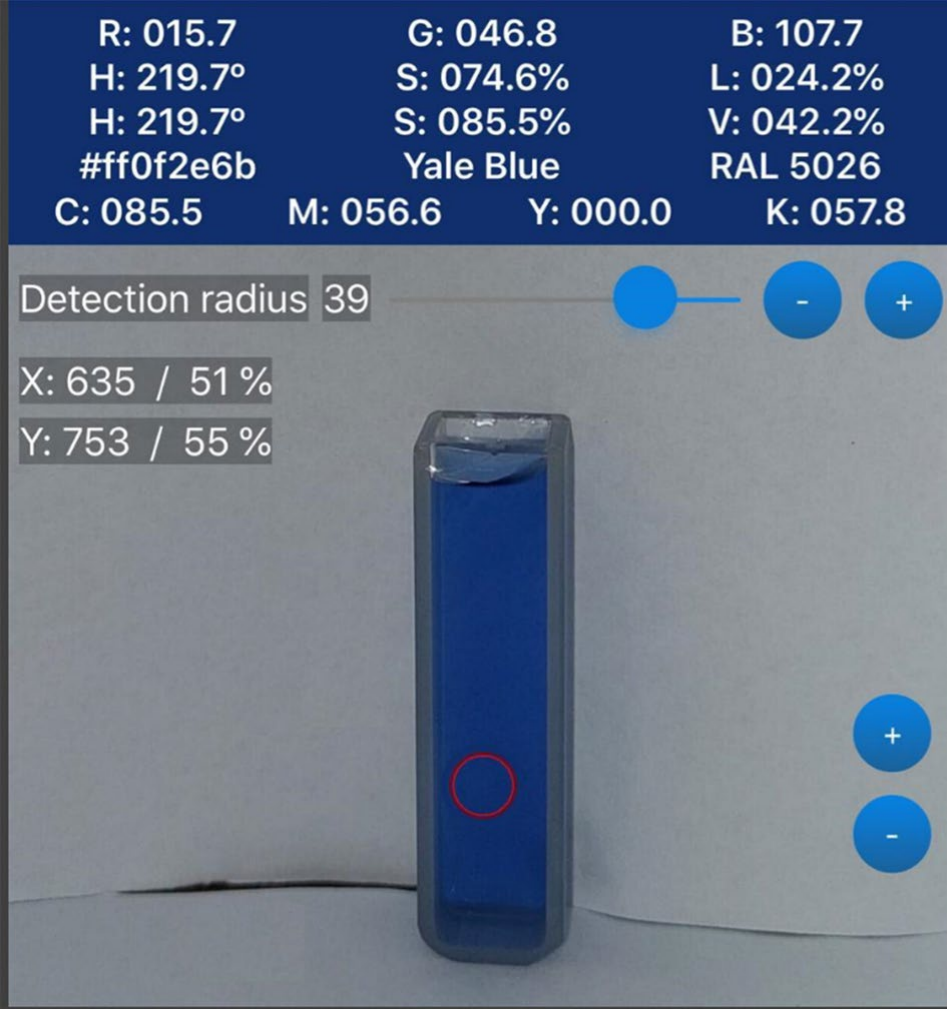
The measurement of color intensity using RGB color detector.

For the assessment of the analytical performance of DIC using Image J, the color quantification was performed using a commonly used mobile application, RGB Color Detector alongside with a spectrophotometer. The mobile application RGB Color detector enables the analysis in four colour spaces—RGB, HSL, HSV, and HSI. The results are generated across eight channels, encompassing R, G, B, H, S, L, V, and I. The measurements were carried out across all channels, and ultimately, channel B was selected for optimal results since the color of the solutions changed from blue to black–blue and the increase in blue values with increasing the blue color intensity was significant Fig. 3.

#### Analytical characteristics of the proposed method

**Figure 4:**
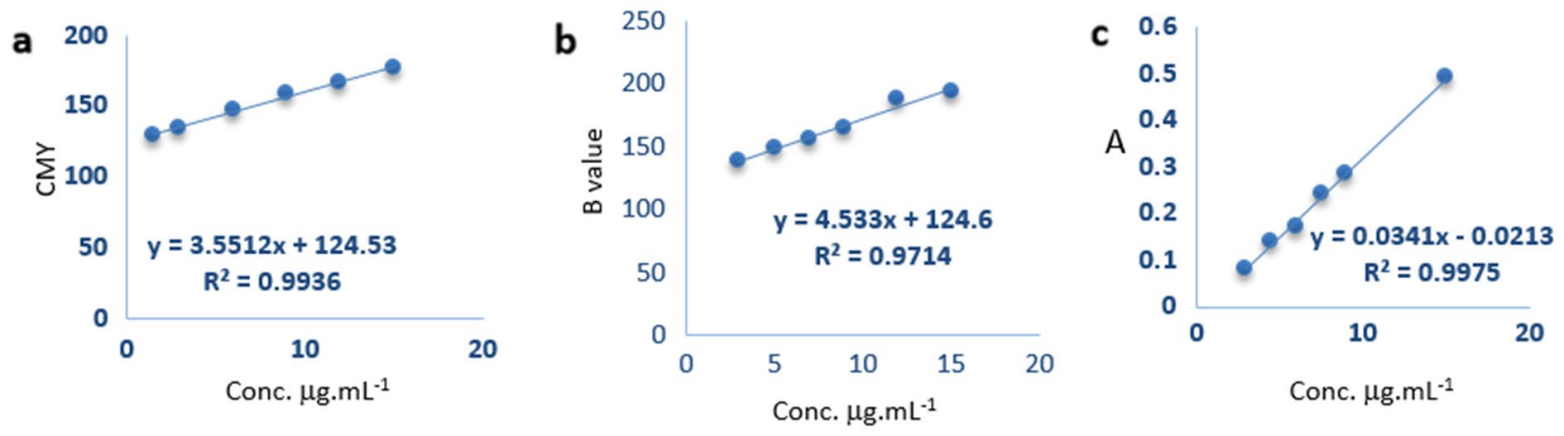
The calibration curves for (**a**) DIC/Image J, (**b**) DIC/RGB, and (**c**) spectrophotometry.

The calibration curve for DIC/Image J was constructed by plotting a relation between the CMY values derived from the images versus the uric acid concentration in diluted artificial urine while, for DIC/RGB analysis, the calibration curve was plotted using B values. For spectrophotometry, the absorbance values at λ_max_ of 700 nm were utilized. Figure S4 shows the absorption spectrum of the blue colored product. The calibration curves were found to be linear over the range 3.0–15 μg·mL^−1^ Fig. 4. The study revealed that the correlation coefficient for DIC/Image J and spectrophotometry is nearly equivalent. However, the correlation coefficient for DIC/RGB showed a lower value of 0.97. The regression equations for all the methods were computed from the corresponding calibration curve. The regression parameters were listed in Table 2.

**Table 2:** Regression parameters for colorimetric determination of uric acid.

Parameters	DIC /Image J	DIC /RGB	Spectrophotometry
Concentration range μg·mL^−1^	3.0–15	3.0–15	3.0–15
Limit of detection LOD μg·mL^−1^	1.199	3.748	0.504
Limit of quantitation LOQ μg·mL^−1^	3.63	11.35	1.53
Regression parameters			
Slope ± SD (Sb)	3.551 ± 0.142	4.533 ± 0.5497	0.034 ± 0.00066
Intercept ± SD (Sa)	124.53 ± 1.29	124.6 ± 5.149	0.0204 ± 0.0052
SD of residual (Sxy)	1.666	4.878	0.00732
Correlation coefficient (r)	0.994	0.9714	0.999

#### Limit of quantitation and limit of detection

The Limit of detection LOD and limit of quantitation LOQ values were calculated according to the following equations:

$$ {\text{LOD}} = {3}.{3}\sigma /{\text{Slope}} $$

$$ {\text{LOQ}} = {1}0\sigma /{\text{Slope}} $$

Where σ is the standard deviation of intercept.

The LOD value achieved with DIC/ Image J was comparable to UV–Vis spectrophotometry, whereas the LOD for DIC/ RGB was apparently higher.

#### Accuracy and precision

The accuracy was verified through the determination of five different concentrations of standard uric acid over the linearity range. The intra-day precision was performed through replicate analysis of five different concentrations of standard uric acid. Inter-day precision was tested by repeated analysis of five different concentrations of uric acid for a period of three successive days. The percent relative error (%RE) was computed for the purpose of method verification and evaluation of the bias between the stated concentration and the measured concentration. It is evident that DIC/Image J exhibit exceptional precision (RSD < 2%) and accuracy (100.39% recovery), comparable to spectrophotometry. On the other hand, the precision and accuracy results from the mobile application are generally lower, yet they remain within an acceptable range. However, at concentrations of 3.0 μg·mL^−1^, the mobile application yielded less accurate and precise results. This indicates that DIC/RGB can be applied adequately for semi-quantitative analysis, while precise and accurate quantitative analysis can be achieved using DIC/Image J. The results of accuracy and precision are listed in Tables 3 and 4.

**Table 3:** Evaluation of the accuracy for determination of uric acid.

Actual Conc. μg·mL^−1^	DIC/Image J			DIC/RGB			Spectrophotometry		
	%Recovery	%RE	Mean %recovery*	%Recovery	%RE	Mean %recovery*	%Recovery	%RE	Mean %recovery*
3	98.116	1.884	100.099	95.09	4.91	98.37	100.83	0.83	100.241
6	101.46	1.46		97.52	2.48		100.38	0.38	
9	100.6	0.61		98.76	1.24		100.07	0.07	
12	100.46	0.46		99.23	0.77		99.954	0.046	
15	99.86	0.14		99.78	0.22		99.97	0.03	

*Average of three determinations.

**Table 4:** Evaluation of the precision for determination of uric acid.

DIC/Image J					DIC/Image J				Spectrophotometry			
Intraday			Interday		Intraday		Interday		Intraday		Interday	
Conc. Taken μg·mL^−1^	Mean Conc. found*	%RSD	Mean Conc. found*	%RSD	Mean Conc. found*	%RSD	Mean Conc. found*	%RSD	Mean Conc. found*	%RSD	Mean Conc. found*	%RSD
3	3.126	1.4	3.198	1.86	2.82	4.87	2.79	5.09	2.99	1.32	3.087	1.54
6	6.208	1.34	6.23	1.73	5.78	2.87	5.75	3.09	6.029	0.958	6.15	1.11
9	9.081	0.857	9.08	0.947	8.86	1.89	8.54	1.36	9.011	0.8	9.093	1.08
12	11.97	0.76	12.23	0.87	12.65	1.74	12.76	1.98	12.102	0.6	11.89	0.95
15	15.09	0.35	15.2	0.45	14.89	1.2	14.56	1.43	15.08	0.26	15.11	0.43

*Average of three determinations.

#### Stability

**Figure 5:**
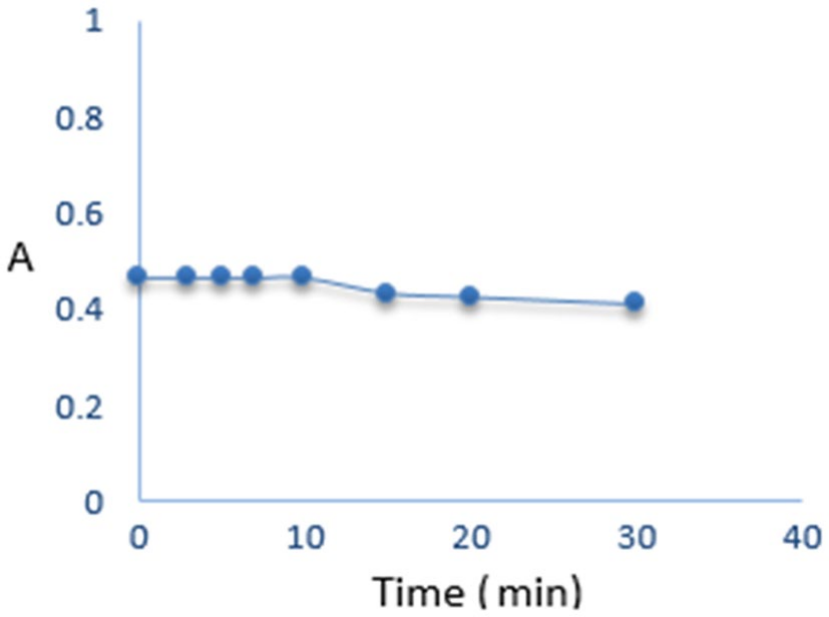
Study of the stability of the blue colored product for 12.0 μg·mL^−1^ uric acid.

The examination of the stability of the blue color formed was conducted over 30 min. The investigation revealed that the blue color remains stable for the initial 10 min, after which its intensity gradually diminishes Fig. 5. Consequently, all measurements were promptly performed immediately following the reaction to get optimal results.

### Standard addition method

The standard addition method was employed for determination of uric acid in real urine to eliminate the matrix effect. The CMY and the absorbance values for the sample were determined from the intercept values of the curves and the concentration was calculated from the regression equations derived from the corresponding calibration curves Figure S5. The results for DIC show that the sample has a concentration of approximately 7.0 μg·mL^−1^ and the initial concentration of 1400 μg·mL^−1^ which were similar to spectrophotometry. This indicates the capability of DIC for accurate determination of uric acid in real samples.

### Greenness assessment

**Figure 6:**
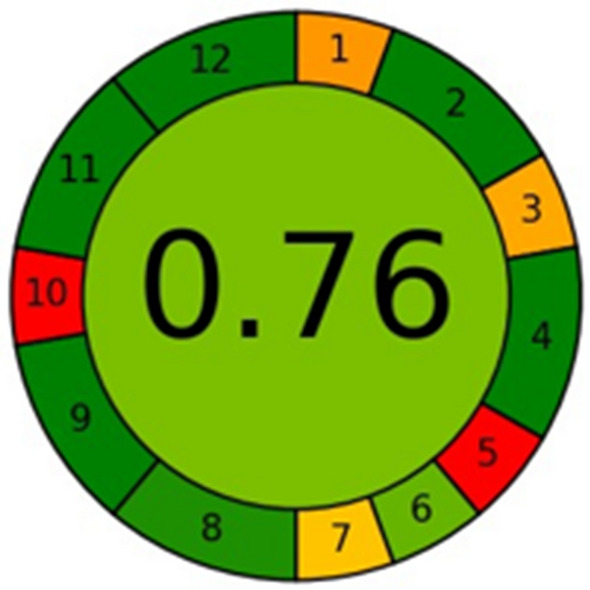
AGREE pictogram for DIC.

The greenness of DIC Method was assessed using Analytical Greenness metric AGREE calculator. This assessment involves 12 principles of green analytical chemistry that are transformed into a standard scale of 0–1 scale reflecting the greenness of the method. The pictogram for AGREE calculator has twelve sections with the total score in the center. The color of each section range from dark green (= 1) to dark red (= 0) based on the ecological impact^37^. The DIC method allows at line and instantaneous analysis of the samples thus it achieved a score of 0.76. Table 5 shows the assessment of AGREE and the adherence of the developed method to the 12 principles of green analytical chemistry, presenting corresponding scores for each principle. Figure 6 represents the pictograms for AGREE metrics for DIC.

**Table 5:** The report for AGREE assessment.

Principle	Comment	Score
Principle 1: Sample Pretreatment Activities	Sample pretreatment such acid deproteinization and treatment with phosphosphotungstate	0.3
Principle 2: Minimal Sample Size	Sample size 10mL	1
Principle 3:In Situ Measurements	At line analysis(little or no sample pretreatment, sample preservation is avoided and preservation agents are not needed	0.33
Principle 4. Integration of Analytical Processes and Operations Saves Energy and Reduces the Use of Reagents	3 steps or fewer	1
Principle 5. Automated and Miniaturized Methods	Manual and not miniaturized	0
Principle 6. Derivatization	Phosphotungstate reagent (CAS 51312-42-6)	0.8
Principle 7. Generation of a Large Volume of Analytical Waste Should Be Avoided and Proper Management of Analytical Waste Should Be Provided	Waste volume = 10 mL	0.39
Principle 8. Multianalyte or Multiparameter Methods	One analyte at a time	0.94
Principle 9. The Use of Energy Should Be Minimized	Non-instrumental detection (smartphone-based analysis)	1
Principle 10. Reagents Obtained from Renewable Source Should Be Preferred	None of the reagents are bio based	0
Principle 11. Toxic Reagents Should Be Eliminated or Replaced	None of the reagents are toxic	1
Principle12. The Safety of the Operator Should Be Increased	None of the reagents are flammable or corrosive	1

### Comparison with other reported colorimetric method

Our research presents a distinctive approach for uric acid determination compared to other reported methods as shown in Table 6. The penalty points (pps) for all reagents and solvents utilized in these methods were calculated as the following^38^.

$$ {\text{The hazard}} = {\text{signal word}}*{\text{number of hazard pictograms}} $$

Where signal word danger = 2pps, warning = 1pps.

**Table 6:** Comparison with other reported colorimetric methods.

Colorimetric method principle	Linearity range	Sample	Detection mode	Comment	Reagents	References
Complexion with potassium ferricyanide, and ferric chloride	2–200 μg·mL^−1^	Urine and serum	Blue colored complex measured at maximum wavelength 752 nm	–	HCl pps = 2 (signal word danger and one hazard pictogram) Potassium ferricyanide pps* = 2 (warning and two pictogram) Ferric chloride pps = 4 (danger and two pictogram)	^31^
MIL-53 Fe as a colorimetric sensor	0.75–10 μg.mL^−1^ (4.5–60 μM)	Urine and serum	Blue colored product measured at maximum wavelength 652 nm	Synthesis of this metal organic frame work requires heating for 17 h and 24 h drying	Solvent DMF pps = 6 (danger and three pictogram) Ferric chloride pps = 4	^32^
AgNPs as a colorimetric sensor	0.0168 ng·mL^−1^ 16.8 μg·mL^−1^ (0.1 nM–0.1 mM)	Spiked serum samples	Color change of the solution from brown to yellow. In parallel, the absorbance is blue shifted from 477 to 428 nm	Synthesis of silver nanoparticles require stirring for 1 h at 95 °C	Silver nitrate pps = 6 (danger and three pictogram)	^33^
WO_3_ NSs as a colorimetric sensor	0.33–30 μg·mL^−1^ (2–180 µM)	Serum	Blue color measured at maximum wavelength 652 nm	Synthesis of nano sheets require magnetic stirring for 72 h and drying for 12 h	Sodium tungsten oxide dihydrate (Na_2_WO_4_·2H_2_O) pps* = 1 (warning and one ictogram) Solvent HNO_3_ pps* = 2 (danger and one pictogram)	^34^
Phosphotungestate	3–15 μg·mL^−1^	Urine	Blue color measured using DIC/Image J	…………	Phosphostungstate reagent pps = 1 (warning and one pictogram)	Our proposed method

The first reported method relies on a complexion reaction involving uric acid, potassium ferricyanide, and ferric chloride in a hydrochloric acid medium HCl which is known for its corrosiveness and irritant properties^31^. Furthermore, two other reported methods involve the utilization of metal–organic framework MIL-53(Fe) and WO_3_ Nanosheets. Despite their high sensitivity, the synthesis of these materials is complex, time-consuming, and necessitates the use of non-green solvents, along with requiring extensive chemical and physical characterization^32,34^. Another method involving silver nanoparticles, while simpler and faster than the former two, still involves multiple procedures, non ecofriendly reagents, and characterization steps^33^. In contrast, our proposed method stands out for its use of green reagents, its one-step reaction process, and non-instrumental determination of color intensity, making it energy-efficient and environmentally friendly. Thus, our approach offers a practical alternative with satisfactory linearity range while addressing the complexities associated with other reported methods.

## Conclusions

This study clearly demonstrates the feasibility of utilizing DIC/Image J for determining uric acid levels in both artificial and real urine samples. This approach offers the advantage of easy monitoring of uric acid without the need for any specialized instruments. Additionally, the use of Image J offers good analytical parameters, at the same time it is uncomplicated and the analysis can be performed directly without the need for numerous trials to determine the optimal channel, as required with mobile APPS. This study also proves that, Image J can be employed for accurate and precise quantitative analysis whereas mobile APPS prove satisfactory for semi-quantitative analysis. Notably this colorimetric approach using Image J has a great potential for various other applications, including colorimetric determination of heavy metals or drug analysis utilizing specific reagents. Overall, this novel method presents a promising avenue for diverse analytical tasks with a smartphone-based approach.

### Supplementary Information

Supplementary Figures.

## Data Availability

All data generated or analyzed during this study are included in this published article (and its Supplementary Information files).
