# Supplementary material for: Smartphone based colorimetric approach for quantitative determination of uric acid using Image J
Source: Sci Rep. 2023 Dec 11;13:21888. doi: 10.1038/s41598-023-48962-0 (PMC10713523; doi:10.1038/s41598-023-48962-0)
Supplement: Supplementary file 1 — Supplementary Figures. [file 41598_2023_48962_MOESM1_ESM.pdf]

Smartphone based colorimetric approach for quantitative determination of uric acid using Image J

Samar H. Elagamy[1]*, Latifa Adly[1] , Mohamed Ahmed Abdel Hamid[1]

1 Department of pharmaceutical analytical chemistry, Faculty of pharmacy, Tanta university, Tanta, Egypt

*Corresponding author Email: samar.elagamy@pharm.tanta.edu.eg

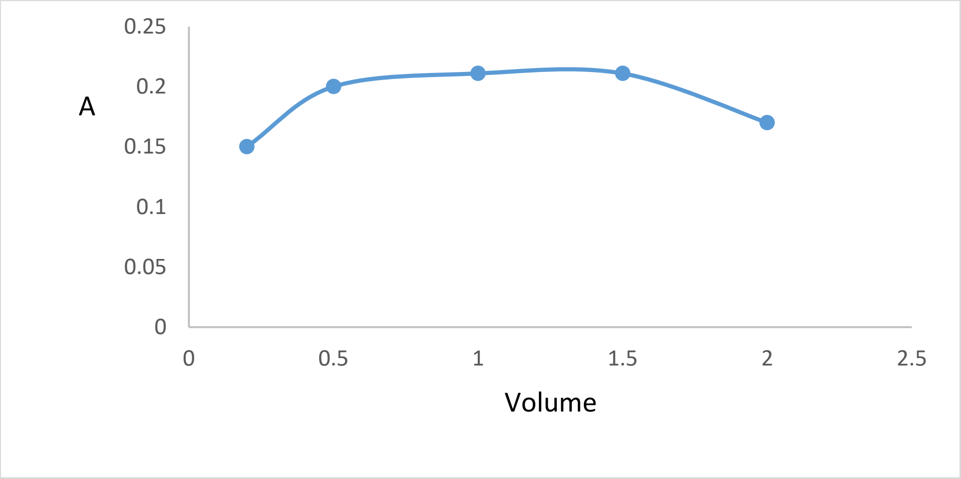

Figure S1: Effect of the volume of phosphotungstate reagent

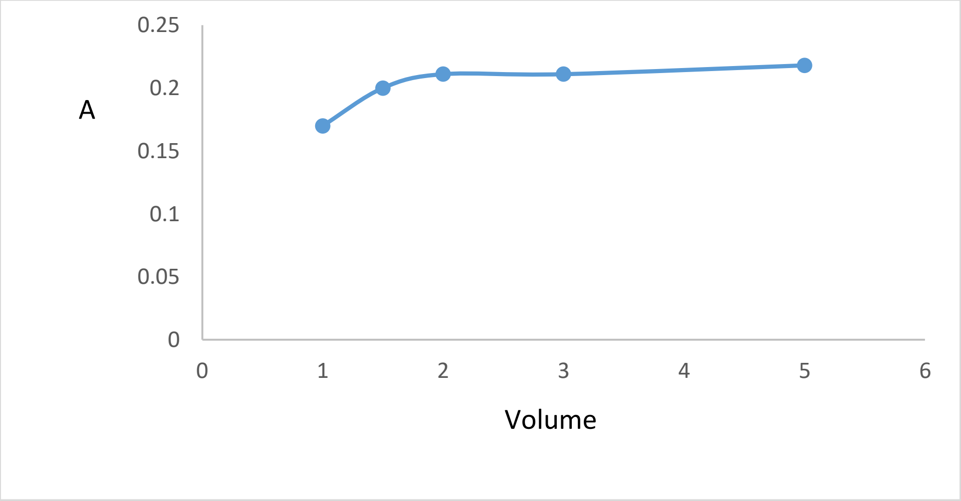

Figure S2: Effect of the volume of sodium carbonate

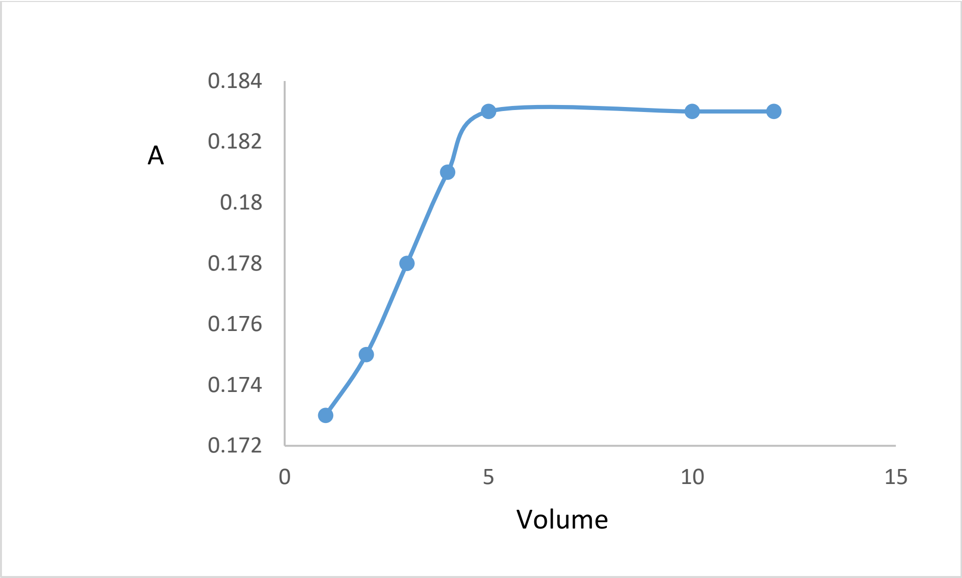

Figure S3: Effect of the volume of vortex time

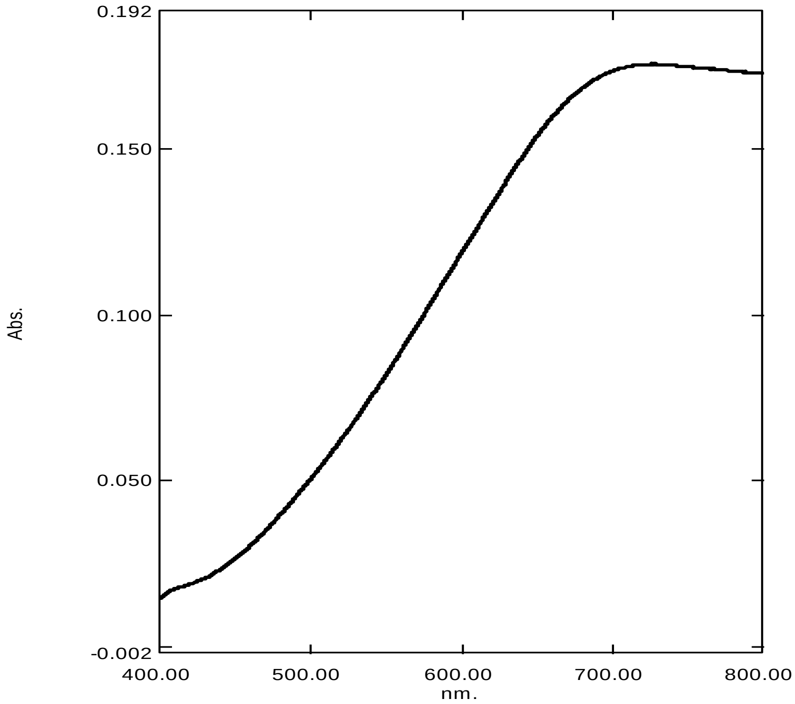

Figure S4: The absorption spectra of the blue colored product after phosphotungstate treatment of 3.0 μg. mL^{-1} uric acid

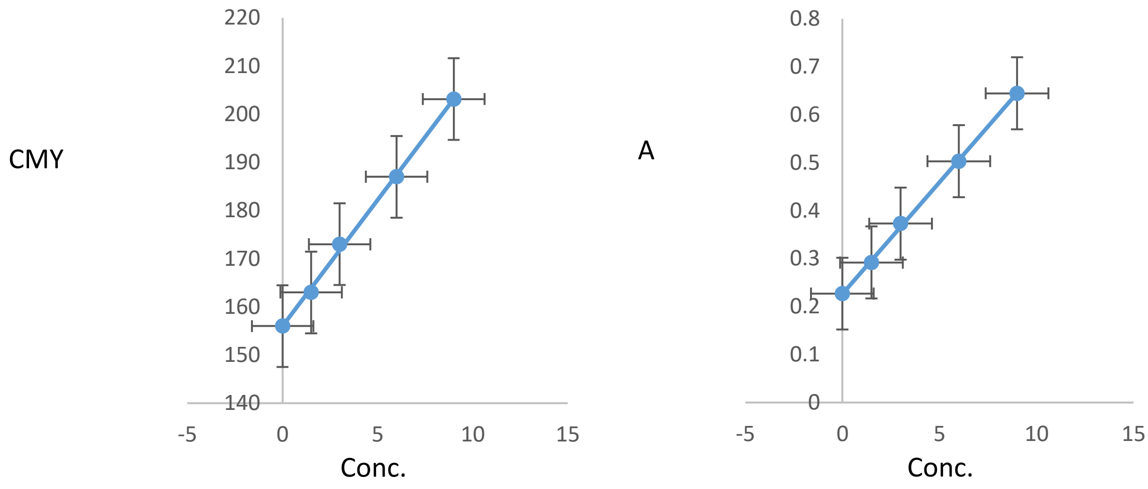

Figure S5 Standard addition plot for DIC (right) and spectrophotometry (left)
